# Comparisons of neurodegenerative disease biomarkers across different biological fluids from patients with Huntington’s disease

**DOI:** 10.1007/s00415-024-12785-4

**Published:** 2025-01-23

**Authors:** Alison R. Bamford, Georgia M. Parkin, Jody Corey-Bloom, Elizabeth A. Thomas

**Affiliations:** 1 Department of Neurobiology and Behavior, University of California Irvine, Irvine, CA USA; 2 Institute for Interdisciplinary Salivary Bioscience Research, University of California Irvine, Irvine, CA USA; 3 Phoenix Australia – Centre for Posttraumatic Mental Health, Department of Psychiatry, University of Melbourne, Parkville, VIC Australia; 4 Department of Neurosciences, University of California San Diego, San Diego, CA USA; 5 Department of Neurosciences, The Scripps Research Institute, La Jolla, CA USA

**Keywords:** Neurodegenerative, Neurodegeneration, Biomarker, Neurofilament, Tau, Saliva

## Abstract

Fluid biomarkers play important roles in many aspects of neurodegenerative diseases, such as Huntington’s disease (HD). However, a main question relates to how well levels of biomarkers measured in CSF are correlated with those measured in peripheral fluids, such as blood or saliva. In this study, we quantified levels of four neurodegenerative disease-related proteins, neurofilament light (NfL), total tau (t-tau), glial fibrillary acidic protein (GFAP) and YKL-40 in matched CSF, plasma and saliva samples from Huntingtin (*HTT*) gene-positive individuals (*n* = 21) using electrochemiluminescence assays. In addition, salivary levels of NfL, t-tau, and GFAP were quantified from a larger cohort (*n* = 95). We found both positive and negative correlations in the levels of these biomarkers among different biofluids. Most notably, in contrast to the significant positive correlations observed between CSF and plasma levels for NfL and GFAP, we detected significant negative correlations between the CSF and saliva levels of NfL and GFAP. With regard to clinical measures, both plasma and CSF levels of NfL were significantly positively correlated with Total Motor Score and chorea, whereas saliva levels of NfL showed significant correlations in the opposite direction. Additional correlations between salivary biomarkers with clinical data, adjusting for age, sex and CAG repeat length, confirmed that salivary NfL was significantly negatively associated with chorea scores in manifest HD, but not premanifest (PM), individuals. In contrast, salivary t-tau was positively associated with measures of cognition in PM participants. These findings suggest that salivary levels of NfL and t-tau proteins may exemplify non-invasive biomarkers for disease symptoms at different stages of illness. Further, these findings highlight the notion that different forms of disease proteins exist in different biological fluids.

**Supplementary Information:**

The online version contains supplementary material available at 10.1007/s00415-024-12785-4.

## Introduction

Biomarkers play a crucial role in multiple aspects of neurodegenerative diseases, such as Huntington’s disease (HD), a fatal, genetic, neurodegenerative disorder, characterized by chorea, motor instabilities, psychiatric manifestations, and cognitive decline [1]. While it is known that carriers of the Huntingtin (*HTT*) gene mutation, an expanded CAG repeat length > 38, will eventually develop HD, there is significant variability in terms of when the disease starts, the severity of symptoms, and how the disease progresses over time [2–5]. The development of biomarkers to track these features may lead to more effective approaches for clinical management of HD, including clinical trial success and overall improved patient care.

Traditionally, cerebrospinal fluid (CSF) has been the gold standard for biomarker measurements, due to its close proximity to brain tissue and its ability to reflect structural and functional changes in the brain. However, less invasive biofluids, such as blood and saliva, are gaining attention due to their practicality and ease of collection, making them attractive alternatives for clinical studies. While blood testing is at the forefront of many recent biomarker studies of neurodegenerative diseases, such as Alzheimer’s disease (AD)—for example, a blood test for p-Tau-217 was recently found to be equivalent to the clinically used FDA-approved CSF tests for AD pathology [6]—saliva represents a completely non-invasive biofluid. Importantly, saliva can be collected in any setting, without trained personnel, and saliva does not require immediate processing. As a diagnostic fluid, saliva has been assessed in a growing number of studies for several systemic conditions, such as celiac disease, rheumatoid arthritis, HIV, diabetes mellitus, and cancer [7–13]; however, it has been underappreciated as a biofluid for CNS-related biomarkers of neurodegenerative diseases.

With regard to correlations of markers across biofluids, several past studies have compared disease biomarkers between CSF and blood, and these studies have shown that levels of two widely studies neurodegenerative disease biomarker, neurofilament light (NfL) and glial fibrillary acidic protein (GFAP), among others, are significantly positively correlated between CSF and blood in patients with neurodegenerative diseases [14, 15]. However, much less is known about how disease biomarkers measured in saliva compare to those found in plasma or CSF.

In this study, we compared levels of NfL and total tau (t-tau), two representative markers of neurons, and GFAP and YKL-40, two markers of astrocytes, in matched CSF, plasma and saliva samples from individuals carrying the *HTT* gene mutation, including premanifest individuals (PM) and those who have already experienced disease onset, or manifest illness (referred to as “HD” patients). In addition, we investigated salivary levels of these biomarkers in a larger cohort of control, PM and HD individuals, along with comparisons to disease data, cognitive measures, and motor symptoms associated with HD.

## Materials and methods

### Participants

This study was approved by the University of California, San Diego (UCSD) Institutional Review Board, in accordance with the requirements of the Code of Federal Regulations on the Protection of Human Subjects. Patients were recruited from the UCSD HDSA Center of Excellence. Premanifest (PM) HD individuals had an *HTT* gene CAG repeat expansion of more than 38 repeats, and a Unified Huntington's Disease Rating Scale (UHDRS) diagnostic confidence rating below 4. Manifest HD patients had a diagnostic confidence rating of 4, indicating that a clinician had ≥ 99% certainty that the patient presented with HD symptoms [16]. Cohort 1 of this study consisted of PM and HD patients who provided matched CSF, plasma, and saliva samples, either on the same day or within 1 day of saliva collection. Cohort 2 consisted of PM and HD patients as well as normal controls who provided only a saliva sample. Normal controls were those with no reported history of neurological conditions, psychiatric disorders, and no use of psychoactive substances. Written consent was obtained from all participants prior to sample collection. Demographic and disease data were obtained at the time of sample collection, including sex, age, CAG repeat length, years of education, and family history.

### Clinical assessments

Clinical assessment included cognitive testing, behavioral and functional measures, and motor ratings. The cognitive battery included the Mini-Mental State Examination (MMSE; score range 0–30) [17], Montreal Cognitive Assessment (MoCA; score range 0–30)[18], Symbol Digit Modalities Test (SDMT; score range 0–110) [19], and Stroop word reading test (SWR). Behavioral and psychiatric changes were assessed using the short form Problem Behaviors Assessment (PBA; maximum score 160) [20]. Functional ability was assessed using the UHDRS [16] Total Functional Capacity (TFC; score range 0–13). Motor dysfunction was evaluated using the UHDRS Total Motor Score (TMS, score range 1–124).

### Biofluid collection

#### Saliva

All donors were asked to refrain from smoking, eating, drinking, or oral hygiene procedures for at least 1 h prior to samples collection. Saliva samples were collected between 10 am and 4 pm using the passive drool method according to previously established protocols [21]. Roughly two milliliters of unstimulated whole saliva was obtained. Samples were immediately frozen at − 20C at the time of collection, then stored at − 80C. At the time of use, saliva samples were thawed and centrifuged (10,000 g; 10 min; 4C) to remove mucins, insoluble material, and cellular debris. Supernatants were collected and used for all assays. Total protein in the saliva supernatants was determined using the BCA protein assay kit (Pierce).

#### Plasma collection

Blood samples from the matched cohort were drawn by venipuncture into 2 ml lavender/EDTA tubes. EDTA/whole blood was mixed well by inversion and spun at 900 g for 15 min. The top plasma layer was transferred into 4 × 1 ml aliquots and frozen and stored at − 80 °C.

#### CSF collection

15–20 ml CSF was collected by lumbar puncture using atraumatic needles (catalog 5181.27; Vygon) and placed immediately onto ice. Collection vessels and plasticware throughout the processing chain were of polypropylene material to minimize protein adsorption. All processing was performed without delay and on ice. CSF was centrifuged at 2,000 g for 10 min to remove cells, divided into aliquots, frozen immediately at –80 °C.

### Biomarker measurements

Levels of GFAP, NfL, t-tau, and YKL-40 were quantified in CSF, plasma, and saliva samples using electrochemiluminescence immunoassay kits from Meso Scale Discovery ([MSD], Gaithersburg, MD, Cat #K15639S, 3-plex for GFAP, NfL and t-tau, and #K151VLK for YKL-40). Both assays are from the MSD U-Plex Assay Platform and wells were first coated with unique biotinylated capture antibodies for 1 h at RT. Plasma and CSF samples were diluted 1:2 in Diluent 12 (MSD), and assays were run according to MSD manufacturers. For saliva, samples were diluted 1:2 in Diluent 12 containing 1X Complete Protease Inhibitor (Sigma-Aldrich) and 1 mM EDTA. For the YKL-40 assay, samples were diluted 1:2 Diluent 57 (MSD). Assays were carried out according to the manufacturer’s protocol, except with an extended incubation time of 2 h. Whenever possible, samples were assayed after a single thaw. On each platform, a single batch of reagents was used for all samples. Measurements were performed in duplicate, and sample measurements accepted if coefficients of variation across duplicates were less than 20%. Detection rates for all biomarkers measured in CSF and plasma were 100%. Detection rate for YKL-40 in saliva was 100%, but other proteins showed lower than 100% detection: t-tau, 98.9%, NfL, 88.4% and GFAP, 81.1%. In addition, the minimum, maximum, mean, and medians levels of all salivary biomarkers can be found in Table S1.

### Statistical analysis

For comparison of cohort characteristics, Pearson’s Chi-squared test was used to compare sex distribution between NC, PM, and HD, whereas one-way analysis of variance (ANOVA) was used to compare age and years of education. Continuous data were tested for normality using the Shapiro–Wilks test and analyzed accordingly using parametric or non-parametric tests. Welch’s two-sample *t* test was used to compare SMDT and Stroop word between PM and HD. And, the Mann–Whitney *U*-test was used to compare CAG repeat length, CAP, MMSE, MoCA, TFC, TMS, chorea, and PBA between PM and HD. Analyte data were tested for normality using the Shapiro–Wilks test and analyzed accordingly using parametric or non-parametric tests. In Cohort 1, the Iglewicz and Hoaglin’s robust test for multiple outliers (two-sided test, Z score ≥ 3.5) detected one outlier for GFAP, three for NfL, two for t-tau, and two outliers in the plasma data, both for YKL-40. In Cohort 2, there were four outliers for GFAP, four for NfL, and two for t-tau; these outliers were removed from all analyses. All correlation analyses were performed using Spearman’s rank correlation or a non-parametric partial correlation test to correct for age or age, sex, and CAG repeat length [22]. Analysis of covariance (ANCOVA) was performed on Cohort 2 data after a natural log transformation to satisfy assumptions for normality of errors and homogeneity of regression slopes. Outlier tests were conducted using Contchart, correlation analyses were performed using IBM SPSS Statistics, correlation matrices and all plots were produced using GraphPad Prism version 10.1, and all other tests were done in Rstudio (R 4.3.3).

## Results

### Participant and biomarker characteristics

Matched CSF, plasma and saliva samples were collected from 21 *HTT* gene-positive individuals, which included both premanifest (PM) and manifest HD patients (Cohort 1). Demographic characteristics are summarized in Table 1. Levels of NfL, t-tau, GFAP, and YKL-40 were quantified in matched biofluids from these subjects and compared demographic (age, sex, years education) and disease-related (CAG length) data. NfL levels in all three fluid were significantly associated with age; however, salivary levels showed a negative correlation with age (*r* = – 0.507, *p* = 0.027), compared to the positive correlations observed for plasma and CSF NfL levels (*r* = 0.53; *p* = 0.016 and *r* = 0.469, *p* = 0.032, for plasma and CSF, respectively) (Table S2). Salivary levels of GFAP were also significantly negatively associated with age, while CSF levels of YKL-40 showed a positive correlation (Table S2). No other correlations between any biomarker and age were detected in any biofluid, nor were any biomarkers associated with sex, nor years of education in any biofluid (Table S2). Further, no biomarkers in any biofluid were significantly associated with the CAG repeat length (Table S2).

**Table 1:** Summary of Cohort 1 participants providing matched CSF, plasma, and saliva samples

	*n*	Mean age (range)	Dx	Mean Edu (yrs)
Males	15	50.0 yrs (39–60)	10PM:5HD	16.8
Females	6	47.2 yrs (38–57)	3PM:3HD	14.8
Total	21	49.2 yrs (38–60)	13PM:8HD	16.2

*PM* premanifest, *HD* manifest patient

### Biomarker correlations across different fluids

**Fig. 1:**
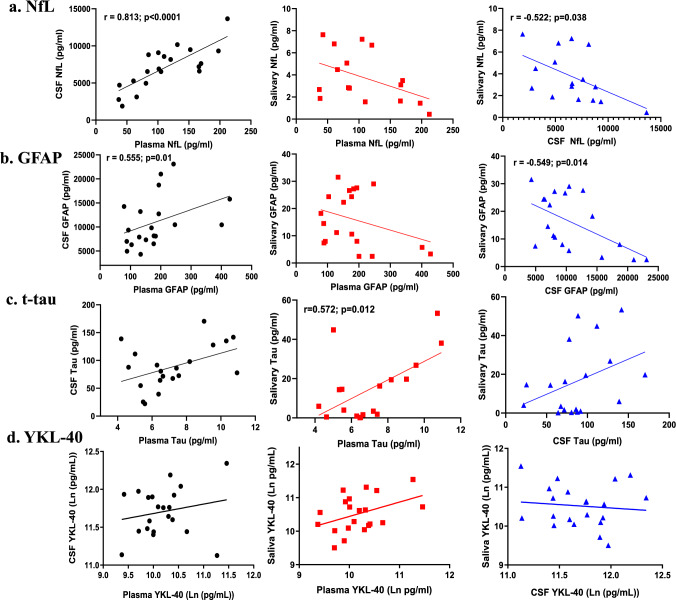
Correlation of NfL, GFAP, t-tau, and YKL-40 across plasma, saliva, and CSF biofluids in Cohort 1. Unadjusted Spearman correlations are shown with the indicated correlation coefficients (rho, r) and *p* values for those correlations that were statistically significant. Data points only reflect values that were above the detection limit of the assay. *NfL* neurofilament light; *GFAP* glial fibrillary acidic protein

**Fig. 2:**
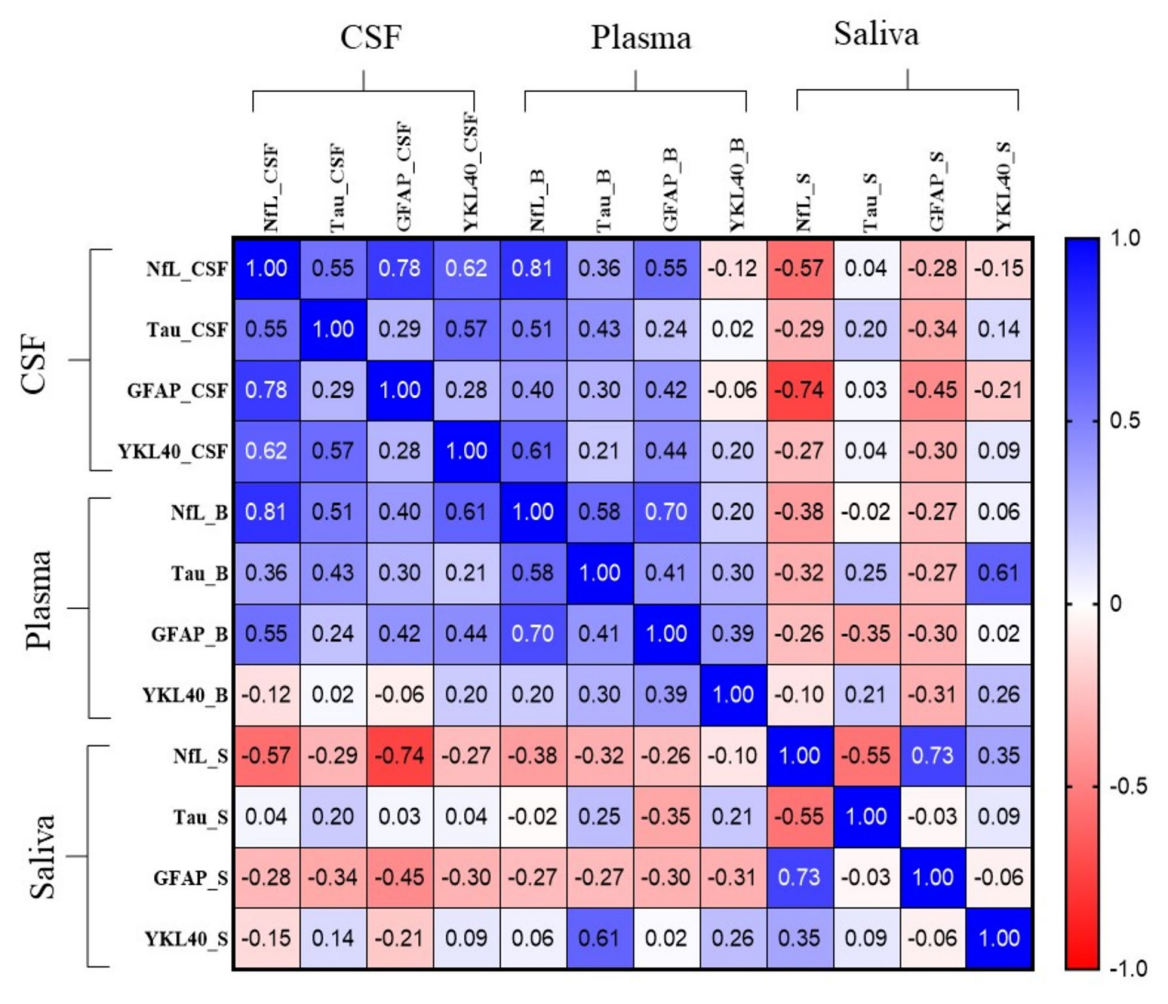
Correlation matrix of neurodegeneration biomarkers across CSF, plasma, and saliva. Correlation matrix reflects unadjusted Spearman correlation analysis. Associated *p* values for these correlations are shown in Table S2. *NfL* neurofilament light; *GFAP* glial fibrillary acidic protein

Comparing biomarker levels between CSF and plasma revealed significant positive correlations for NfL and GFAP (*r* = 0.743, *p* < 0.001 and *r* = 0.489, *p* = 0.029, for NfL and GFAP, respectively; Fig. 1). Levels of t-tau and YKL-40 were not significantly correlated between CSF and plasma samples (Fig. 1). Comparing biomarker levels between plasma and saliva samples showed positive associations for t-tau and YKL-40, although the latter correlation did not reach statistical significance (Fig. 1). In contrast to these positive associations, negative correlations were detected between plasma and saliva for NfL and GFAP, although neither association reached statistical significance (Fig. 1). Significant negative correlations were observed for NfL and GFAP (*r* = – 0.522, *p* = 0.038 and *r* = – 0.550, *p* = 0.015, for NfL and GFAP, respectively), but no significant correlations for t-tau and YKL-40 between CSF and saliva (Fig. 1). Comparing salivary biomarkers to the total protein in each sample revealed only a significant correlation for YKL-40 (*r* = 0.636; *p* = 0.003). In addition, we observed significant correlations among different biomarkers across the biofluids, as reflected by a heatmap showing the correlation coefficients (Fig. 2; corresponding *p* values for these correlation are provided in Table S3). For example, in addition to demonstrating that all biomarkers in the CSF were highly intercorrelated, we found that CSF levels of GFAP were correlated with salivary levels of NfL, and plasma levels of t-tau with saliva levels of YKL-40 (Fig. 2; Table S3).

### Biomarker correlations to clinical data

We next compared biomarker levels in each biofluid with a focused set of clinical measures, including MoCA, SDMT, TFC, TMS, and chorea. Because only age showed a significant association with any of the biomarkers, Pearson correlations were adjusted for only age. Results revealed significant unadjusted correlations for NfL and t-tau versus the motoric symptoms, TMS and chorea, in all biofluids tested and these association largely remained after adjusting for age, with the exception of CSF correlations, which were slightly weakened (Table 2). Correlations for NfL and t-tau also remained when saliva values were normalized to total protein (data not shown). GFAP was found to be negatively correlated with TFC in plasma samples (*r* = – 0.513, *p* = 0.021) and with MoCA in saliva samples (*r* = – 0.539, *p* = 0.047, after age adjustment), while YKL-40 was not significantly associated with any clinical measure in any biofluid (Table 2), hence was not further studied.

**Table 2:**
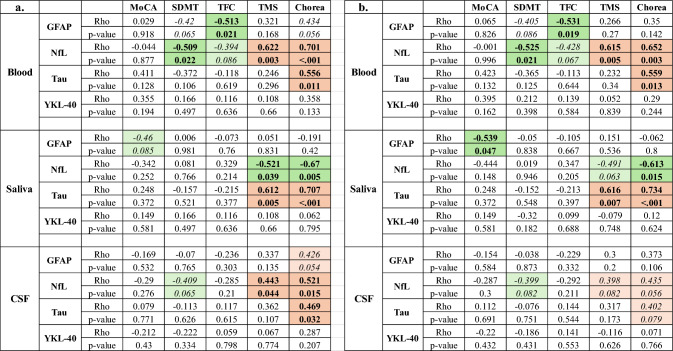
Unadjusted (a) and age-adjusted (b) correlations between biomarkers and clinical data in CSF, plasma, and saliva samples from *HTT* gene-positive patients from Cohort 1

Significant correlations at unadjusted *p* < 0.05 are shown in bold, with those associations close to significance (*p* < 0.1) shown in italics. Colored cells indicate the direction of the correlations, with green depicting negative correlations and pink depicting positive correlations

*MoCA* Montreal Cognitive Assessment; *MMSE* Mini-Mental State Examination; *SDMT* Symbol Digit Modalities Test; *TFC* Total Functional Capacity; *TMS* Total Motor Score; *PBA* Problem Behaviors Assessment

### Validation of saliva–plasma NfL correlation in a larger HD cohort

**Fig. 3:**
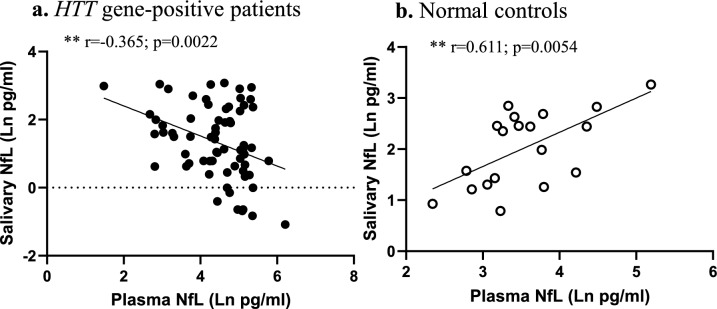
Correlations between plasma and salivary levels of NfL in HD mutation carriers (**a**) and normal controls (**b**). Spearman correlation analysis was carried out on gene-positive (PM plus HD) individuals (*n* = 68) and normal controls (*n* = 20). Data points only reflect values that were above the detection limit of the assay

We further measured saliva levels of NfL, t-tau, and GFAP in a larger cohort of PM and HD patients (*n* = 75) as well as normal controls (*n* = 20) (Cohort 2). A summary of the demographic and clinical data from these samples is shown in Table 3. Cohorts differed significantly by age; HD and NC cohorts were significantly older than PM, and differed in their sex ratios with NC and PM cohorts having more males, and HD patients more females. HD patients also had significantly worse mean symptom counts for several clinical measures compared to the PM cohort (Table 3). For comparisons of salivary and plasma NfL, we retrieved plasma NfL data from our previous study [23]. Similar to the findings on the smaller matched cohort above, we found that plasma levels of NfL were significantly negatively correlated with saliva levels in HD gene-positive individuals (PM plus HD patients) (*r* = – 0.365, *p* = 0.0022; Fig. 3). In contrast, plasma and salivary levels of NfL were positively correlated in the subset of normal control subjects (*r* = 0.611, *p* = 0.0054; Fig. 3). Plasma data for GFAP and Tau were not available on this larger cohort, so additional comparisons could not be made.

**Table 3:** Summary of Cohort 2 demographic information and associated clinical measures

	NC	PM	HD	*p *value
Sex (F:M)	20 (8:12)	35(12:23)	40 (25:15)	**0.0385**
Age	50.4 yrs (13.1)	43.9 yrs (10.8)	57.0 yrs (12.0)	**3.32E-05**
Education	15.1 (2.58) yrs	15.7 (2.81) yrs	15.4 (3.13)yrs	0.798
CAG repeat	NA	41.8 (2.2)	42.5 (2.3)	0.263
MMSE	NA	28.03 (2.13)	25.89 (3.46)	**0.00412**
MoCA	NA	27.44 (2.64)	23.51 (6.11)	**6.88E-04**
SDMT	NA	49.34 (13.0)	31.23 (14.03)	**1.98E-07**
TFC	NA	12.49 (1.15)	9.9 (2.73)	**9.95E-08**
TMS	NA	1.77 (2.37)	26.85 (15.89)	**1.38E-13**
Chorea	NA	0.34 (0.80)	5.87 (4.03)	**2.13E-13**
PBA	NA	4.14 (5.46)	9.66 (9.48)	**0.00482**

The mean value and standard deviation (S.D) are shown

*PM* premanifest; *MoCA* Montreal Cognitive Assessment; *MMSE* Mini-Mental State Examination; *SDMT* Symbol Digit Modalities Test; *TFC* Total Functional Capacity; *TMS* Total Motor Score; *PBA* Problem Behaviors Assessment

Significant associations are shown in bold

### Correlations of salivary biomarkers with clinical data in the larger cohort

Given the significant differences in disease symptoms between PM and HD participants, we assessed associations between salivary biomarkers and clinical measures in PM and HD cohorts separately. In PM individuals, we detected significant positive associations between salivary t-tau and MoCA, MMSE, and TFC scores (Table 4); however, only correlations with TFC remained significant after adjusting for age, sex, and CAG repeat length (Table 4). Significant unadjusted correlations between salivary t-tau and MoCA and MMSE were also observed in HD individuals, but these were no longer significant after adjustment for age, sex, and CAG repeat length (Table 4). Similar to our findings on the smaller matched cohort, salivary levels of NfL were negatively associated with chorea symptoms, but only in HD patients (Table 4), and this effect persisted after adjusting for age, sex, and CAG repeat length (Table 4). Correspondingly, salivary GFAP levels were associated with PBA symptoms only in HD patients and after adjusting for covariables (Table 4).

**Table 4:**
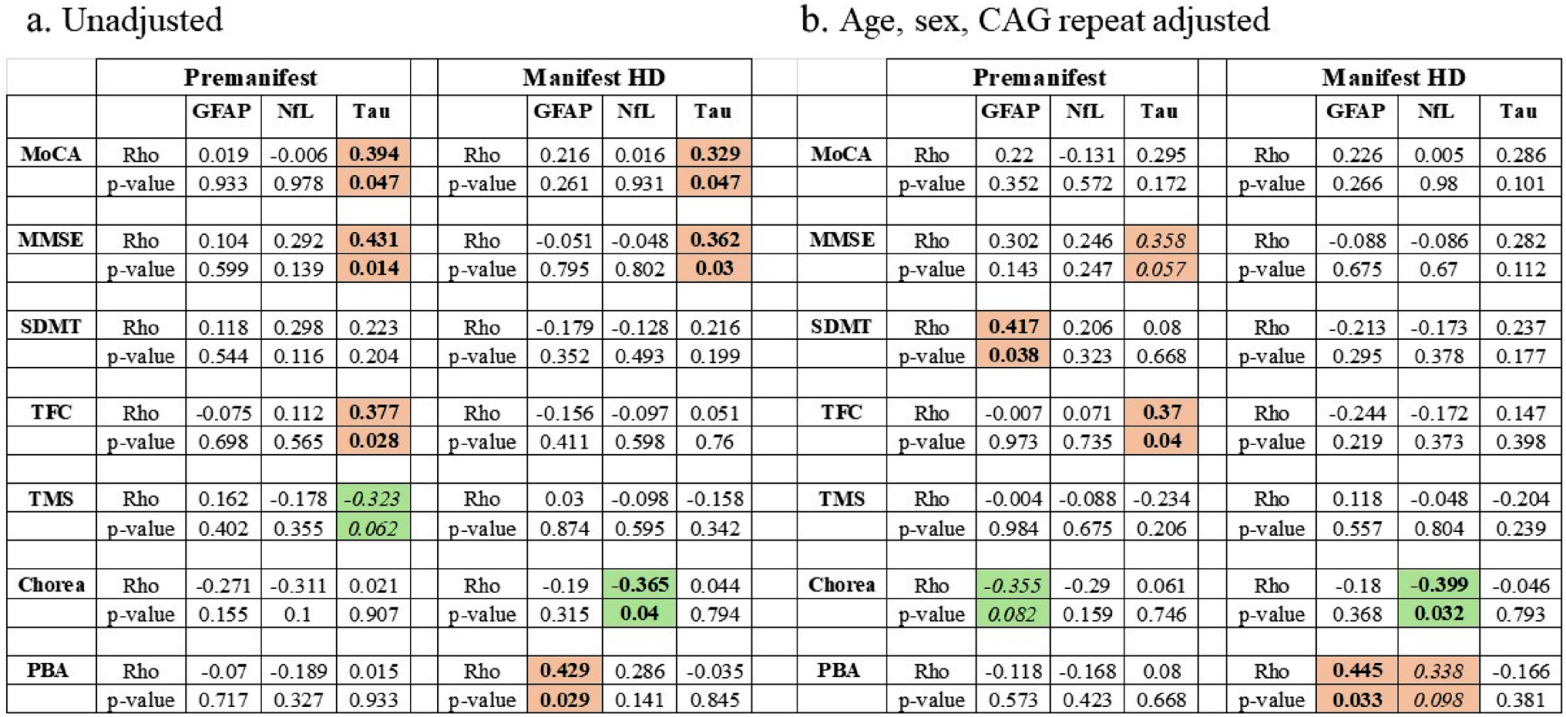
Comparison of unadjusted (a.) and age, sex, and CAG repeat length adjusted (b.) correlations of salivary biomarkers between PM and HD patients

Unadjusted correlations reflect Spearman correlation analysis (Panel a). Non-parametric partial correlations were carried out adjusting for age, sex, and CAG repeat length (Panel b). For PM individuals, the numbers of biomarkers above the detection limit, and therefore included in the analyses, were *n* = 29 for GFAP and NfL and *n* = 34 for Tau. For HD patients, the numbers were *n* = 27 for GFAP, *n* = 29 for NfL and *n* = 35 for Tau. Significant correlations at unadjusted *p* < 0.05 are shown in bold, with those associations close to significance (*p* < 0.1) shown in italics. Colored cells indicate the direction of the correlations, with green depicting negative correlations and pink depicting positive correlations

*MoCA* Montreal Cognitive Assessment; *MMSE* Mini-Mental State Examination; *SDMT* Symbol Digit Modalities Test; *TFC* Total Functional Capacity; *TMS* Total Motor Score; *PBA* Problem Behaviors Assessment

### Comparisons of salivary biomarkers across diagnostic groups

**Fig. 4:**
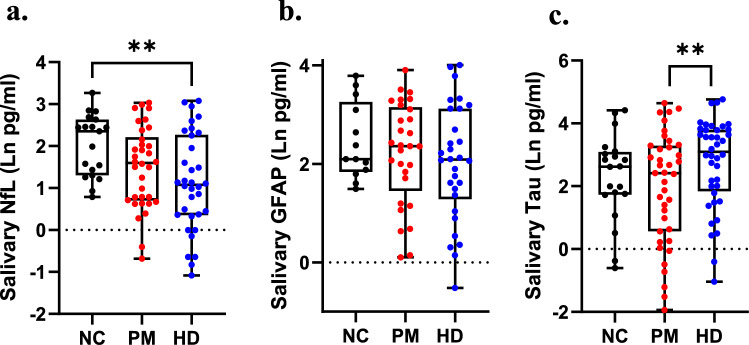
Group-wise comparisons of salivary levels of NfL (**a**), GFAP (**b**), and t-tau (**c**) across different diagnostic groups. Statistical comparisons reflect ANCOVA adjusting for age across normal control (NC), premanifest (PM) and HD diagnoses. Asterisks reflect post hoc comparisons **, *p* < 0.01

Comparing salivary biomarker levels across diagnostic groups using ANCOVA adjusting for age and sex showed significant differences in salivary NfL and t-tau levels across NC, PM, and HD diagnoses (F(2,76) = 5.25; *p* = 0.0072 for NfL and F = (2,88) = 4.31; *p* = 0.016, for t-tau). Post hoc tests showed a significant difference between NC and HD individuals for NfL (*p* = 0.008) and between PM and HD individuals for t-tau (*p* = 0.003) (Fig. 4). Salivary levels of GFAP were not different according to diagnostic group (Fig. 4).

## Discussion

In this study, we highlight associations between the widely studied neurodegenerative disease biomarkers, NfL, t-tau, GFAP and YKL-40, across three biological fluids in *HTT* gene-positive patients. Despite the small size our of Cohort 1, we were able to robustly recapitulate the known correlations observed between CSF and plasma for NfL and GFAP [14, 15]. We did not detect significant correlations between CSF and plasma for t-tau, which is also consistent with previous studies [24]. However, we also demonstrate previously unreported associations between commonly studied CNS biomarkers across different biofluids in HD patients, namely significant negative correlations between CSF and saliva for NfL and GFAP.

NfL is one of the most frequently studied fluid biomarker in HD [25–29], mainly due to its exclusive expression in neurons, where it serves as a major structural component. Under physiological conditions, there is a basal release of NfL into the CSF, which is known to increase with age [30]. However, its release greatly increases after axonal damage in neurodegenerative conditions and other conditions of brain injury or stroke [31–33]. As mentioned above, significant associations have previously been reported between either plasma or serum NfL and those levels measured in the CSF, for a number of CNS disorders [34–37], making it a reliable biomarker for tracking disease progression and response to treatment. Accordingly, plasma levels of NfL are being used in HD clinical trials as an exploratory biomarker for assessing therapeutic efficacy. However, recent studies have revealed that different forms of NfL exist in the brain and CSF and in neurodegenerative conditions, shedding some light on the actual molecular state of this protein in the brain and biological fluids.

Using immunoprecipitation–mass spectrometry analysis, Budelier and colleagues showed that no full-length NfL was present in the CSF, but rather NfL existed as various truncated species [38]. This was in contrast to the full-length version of NfL that is found in human brain [38]. Additional studies using size-exclusion chromatography confirmed these findings and further reported that NfL exists largely as oligomers in the CSF [39]. Furthermore, this study showed that oligomeric NfL was higher in patients with primary progressive aphasia and AD compared to normal controls, in which oligomeric NfL was not abundantly found [39]. With the breakdown of the blood–brain barrier in neurodegenerative states, it could be assumed that oligomeric NfL is also present in the blood. It is also known that some proteins are transported from the blood into the saliva by different transport mechanisms depending on the biomarker [40, 41]. Hence, one possible explanation of the negative correlation we observed between salivary and CSF/plasma levels of NfL is that oligomeric species of NfL cannot be easily transported into saliva. This hypothesis is also supported by our findings from Cohort 2, where we validated a negative correlation between plasma and saliva NfL in HD gene-positive individuals, but observed a positive correlation between these two fluids for NfL in normal control subjects, whereby oligomeric NfL is not readily found.

Like NfL, we found that GFAP levels in saliva were also negatively correlated with those found in CSF. GFAP is a cytoskeletal monomeric filament protein present in astroglial cells in the brain [42]. GFAP has been shown to exist in multiple isoforms in the brains of patients with AD [43, 44] and specifically can form oligomers in human astrocytes [45]. GFAP also exhibits post-translational modifications [46], which could affect transport across different biological fluids. GFAP has been previously measured in saliva and one study has reported that salivary GFAP is reduced in demented patients compared to healthy donors [47]; this effect is opposite to what has been shown for plasma GFAP [48, 49]. This same study also showed that GFAP in saliva from AD patients was mainly of higher molecular weights, while many different GFAP isoforms were present in the saliva from healthy donors [47]. Again, it could be hypothesized that these modifications may impair the ability of GFAP to translocate into the saliva, thus explaining the observed negative associations between saliva and CSF.

The negative correlation observed between CSF/plasma and saliva levels of NfL was also consistent with the negative association we observed between salivary NfL and TMS and chorea symptoms in Cohort 1, whereby on the other hand, positive correlations were detected between CSF/plasma levels of NfL and these motoric symptoms, consistent with past studies [23, 28, 29, 50]. The negative correlations observed between salivary NfL and chorea were also observed in the larger Cohort 2, but only in HD patients or when PM and HD individuals were combined (data not shown). The fact that salivary NfL was not correlated with chorea in PM subjects could indicate that during the premanifest period, NfL is in a mixed molecular state consisting of monomers and various forms of oligomers. Accordingly, in manifest illness, it may be that more NfL is in the oligomeric form, similar to the studies reported in AD [39], consequently, less is present in saliva. Accordingly, it is possible that salivary NfL represents a better signal of neurodegeneration after disease onset, as it is specifically not repressing the multimer forms, whereas measurements in plasma and CSF would reflect all forms.

Our current studies also demonstrate significant correlations between plasma and salivary levels of t-tau protein, suggesting that salivary t-tau may hold promise as a non-invasive biomarker. Like NfL, t-tau has emerged as a biomarker for cognition in several neurodegenerative diseases, even HD, with levels correlating with several aspects of cognitive deficits [51, 52]. Tau is a microtubule-associated protein which exists in six different isoforms in the healthy adult human brain, and has many post-translationally modified forms. Our findings show positive associations between t-tau present in all three biofluids with chorea symptoms in Cohort 1. However, we did not validate this finding for salivary t-tau in our larger Cohort 2, when patients were separated according to premanifest or manifest stages. Rather, we found significant associations between salivary t-tau and cognitive tests, such as MMSE and MoCA. These findings were more consistent in PM subjects compared to HD. Although, t-tau was not significantly different in HD gene-positive patients compared to normal controls, we did detect a significant difference between PM and HD patients (Fig. 4).

Comparing NfL across diagnoses, we found that salivary levels were lower in PM and HD patients compared to normal controls. One previous study has investigated salivary levels of NfL in two mixed memory clinic cohorts, including AD, mild cognitive impairment (MCI), non-AD dementia, and healthy controls [53]. In that study, no statistically significant differences were found in salivary NfL concentration across the diagnostic groups [53]. This study also did not report any significant correlations between plasma and saliva NfL levels; however, upon visual inspection of their data from Fig. 2 of the paper, it is clear that there is a negative association between plasma and salivary NfL levels in AD and non-AD dementia patients, albeit not statistically significant, but not in healthy controls, nor in MCI patients [53]. This would be consistent with our findings of a negative correlation between plasma and saliva NfL levels in PM and HD patients, but not in normal controls (Fig. 3).

## Conclusion

The use of saliva for biomarker research in neurodegenerative diseases has been growing in past years, with previous studies already focused on its potential utility in HD [41, 54–56], including our previous study showing that the huntingtin protein is uniquely processed in saliva, compared to blood [41]. Our current studies suggest that NfL and GFAP may also exist in different molecular forms in saliva compared to plasma and CSF, although further studies using Western blot analysis or mass spectrometry would be needed to confirm this hypothesis. Nonetheless, we suggest that salivary levels of t-tau and NfL could serve as non-invasive biomarkers, representing early (PM) and later (manifest HD) stages of illness, respectively, and provide new information regarding the development and disease progression HD.

## Supplementary Information

Below is the link to the electronic supplementary material.

Supplementary file1 (DOCX 32 KB)

## Data Availability

Anonymized summary data will be shared by reasonable formal request from qualified researchers, subject to a data sharing agreement and in compliance with the requirements of the funding bodies and institutions.
