# Supplementary material for: Comparisons of neurodegenerative disease biomarkers across different biological fluids from patients with Huntington’s disease
Source: J Neurol. 2025 Jan 23;272(2):158. doi: 10.1007/s00415-024-12785-4 (PMC11759467; doi:10.1007/s00415-024-12785-4)
Supplement: Supplementary file 1 — Supplementary file1 (DOCX 32 KB) [file 415_2024_12785_MOESM1_ESM.docx]

**Supplementary Information**

**Table S1. Summary of NfL, Tau, YKL-40 and GFAP measurements in saliva, plasma and CSF samples from Cohort 1.**

**Table S2. Correlations between biofluid markers and age, sex, education and CAG repeat length.**

**Table S3. Summary of salivary measures of GFAP, NfL and t-tau from Cohort 2.**

**Table S4. P-values from the correlation matrix shown in Figure 2.**
